# Development and validation of ischemic heart disease and stroke prognostic models using large-scale real-world data from Japan

**DOI:** 10.1265/ehpm.22-00106

**Published:** 2023-02-15

**Authors:** Shigeto Yoshida, Shu Tanaka, Masafumi Okada, Takuya Ohki, Kazumasa Yamagishi, Yasushi Okuno

**Affiliations:** 1 Data Science and Advanced Analytics, IQVIA Solutions Japan K.K., 4-10-18 Keikyu Dai-ichi Bldg., Takanawa, Minato-ku, Tokyo 108-0074, Japan; 2 Real-World Evidence Solutions, IQVIA Solutions Japan K.K., 4-10-18 Keikyu Dai-ichi Bldg., Takanawa, Minato-ku, Tokyo 108-0074, Japan; 3 Department of Public Health Medicine, Faculty of Medicine, and Health Services Research and Development Center, University of Tsukuba, 1-1-1 Tennodai, Tsukuba 305-8575, Japan; 4 Medical Sciences Innovation Hub Program, RIKEN, 1-7-22 Suehiro-cho, Tsurumi-ku, Yokohama 230-0045, Japan; 5 Graduate School of Medicine, Kyoto University, Sakyo-ku, Kyoto 606-8507, Japan

**Keywords:** Risk prediction model, Machine learning, Ischemic heart disease, Stroke, Real-world data

## Abstract

**Background:**

Previous cardiovascular risk prediction models in Japan have utilized prospective cohort studies with concise data. As the health information including health check-up records and administrative claims becomes digitalized and publicly available, application of large datasets based on such real-world data can achieve prediction accuracy and support social implementation of cardiovascular disease risk prediction models in preventive and clinical practice. In this study, classical regression and machine learning methods were explored to develop ischemic heart disease (IHD) and stroke prognostic models using real-world data.

**Methods:**

IQVIA Japan Claims Database was searched to include 691,160 individuals (predominantly corporate employees and their families working in secondary and tertiary industries) with at least one annual health check-up record during the identification period (April 2013–December 2018). The primary outcome of the study was the first recorded IHD or stroke event. Predictors were annual health check-up records at the index year-month, comprising demographic characteristics, laboratory tests, and questionnaire features. Four prediction models (Cox, Elnet-Cox, XGBoost, and Ensemble) were assessed in the present study to develop a cardiovascular disease risk prediction model for Japan.

**Results:**

The analysis cohort consisted of 572,971 invididuals. All prediction models showed similarly good performance. The Harrell’s C-index was close to 0.9 for all IHD models, and above 0.7 for stroke models. In IHD models, age, sex, high-density lipoprotein, low-density lipoprotein, cholesterol, and systolic blood pressure had higher importance, while in stroke models systolic blood pressure and age had higher importance.

**Conclusion:**

Our study analyzed classical regression and machine learning algorithms to develop cardiovascular disease risk prediction models for IHD and stroke in Japan that can be applied to practical use in a large population with predictive accuracy.

**Supplementary information:**

The online version contains supplementary material available at https://doi.org/10.1265/ehpm.22-00106.

## Graphical Abstract

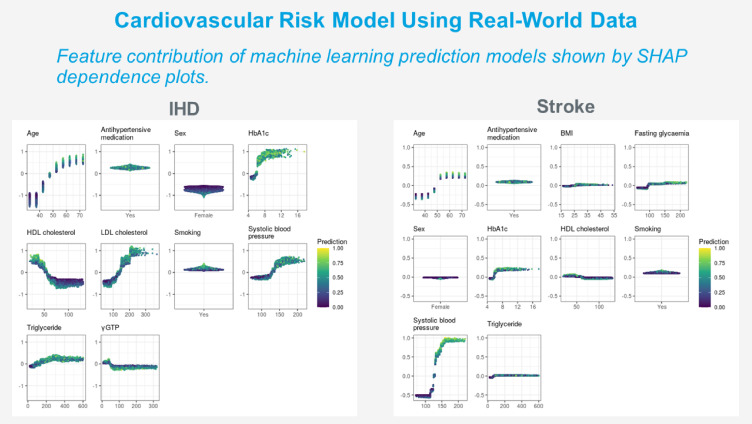

## Introduction

Cardiovascular disease continues to be the leading cause of death globally, causing nearly 9 million deaths in 2019, with ischemic heart disease (IHD) and stroke accounting for most global deaths [1]. Compared to Western Europe, North America, South America, and Australia, stroke mortality was higher in Asia with some exceptions in East Asia [2]. Risk assessment is an effective strategy for cardiovascular disease prevention, and advanced risk prediction tools have been developed to predict the future risk for cardiovascular disease [3, 4].

In Japan, cardiovascular disease risk prediction models have been developed in the past with data from several prospective cohort studies [5–8]. Such cohort studies obtain concrete epidemiological evidence in target populations, but often last for decades. In contrast, real-world data (RWD), especially medical big data, enables development of disease risk prediction models based on larger datasets, and it potentially allows to develop prediction models targeting different outcomes in a shorter time span than the prospective cohort studies. Furthermore, machine learning (ML) technologies can take advantage of digitalized nature of RWD, such study framework will facilitate faster implementation and refinement of disease risk prediction models in clinical practice.

Administrative social health insurance claims data in Japan involve individuals aged below 65 years, and the enrollees take an annual health check-up involving physical measurements, blood tests, urine and fecal tests, and a questionnaire regarding the enrollee’s medical history, health condition, and lifestyle [9]. The findings of the annual health check-up can be applied to prognostic model development, especially for the working population, and further combined with RWD to perform large-scale risk prediction analysis applicable to the broader population.

Traditional regression models such as logistic or Cox proportional-hazards (Cox) are extensively used for prognostic models. Cox is a linear method and assumes that the hazard rate is linearly related to the risk factors. Extreme gradient boosting (XGBoost) involves the implementation of boosted decision trees that furnish prediction results through a repetitive process of prediction summation in decision trees. The combination of many trees in the XGBoost model promotes nonlinearity and interactions between features [10]. XGBoost has been utilized in medical research in recent years [11].

The present study aims to develop IHD and stroke prognostic models using large-scale RWD by statistical and ML procedures, with a focus on social implementation. This study determines the risk prediction rather than investigating a causal relationship between features and outcomes. It further evaluates model performance and examines the contribution of relevant features to model prediction.

## Methods

### Data source

The cohort of study participants was derived from Japan administrative claims data provided by IQVIA Solutions Japan K.K. (Tokyo, Japan) (hereinafter referred to as IQVIA Claims Database) [12]. The IQVIA Claims Database comprised of anonymized data for approximately 3.5 million individuals corresponding to 2.8% of population and 8.4% of social health insurance coverage in Japan. Age distribution of the database was almost the same as the distribution of the Japanese population between ages 0–64 years, but the database included only a few percent of individuals aged 65 and over. Majority of individuals in the database were corporate employees and their families in the secondary and tertiary industries distributed across Japan, while individuals belonging to the primary industry, employees of local medium-to-small sized companies, and self-employed workers were rarely included [12]. Information in the IQVIA Claims Database was derived from administrative claims and annual health check-up data provided by health insurance providers, and included tables of basic demographics, diagnosis, medication, treatment, and annual health check-up records of employees and family members from April 2013. Tables in the database were linked by an anonymized individual identifier. Documented diagnosis was specified as the standard disease mapping system in Japan (Medical Information System Development Center), which is consistent with the International Classification of Diseases, 10^th^ Revision (ICD-10).

### Study cohort

The study population encompassed individuals from the IQVIA Claims Database with at least one annual medical health check-up record between April 2013 and December 2018 (identification period), and aged 30 to 74 years at the first annual medical health check-up (index year-month) during the identification period. To reduce the computation time, 50% randomly sampled individuals were included in the study. Random sampling was performed by randomly extracting 50% of individual’s unique identifiers from a table containing membership information in the database. Individuals with a history of cancer (ICD-10: C00.x–C26.x, C30.x–C41.x, C43.x–C58.x and C60.x–C97.x) before the index year-month were also excluded from the analysis, considering any possible difference in characteristics from rest of the population. Individuals with a history of stroke or IHD before the index year-month were excluded from the analysis. Furthermore, disqualified individuals at the index year-month were also excluded from the analysis. Study participants were followed up from index year-month until the event onset or diagnosed with cancer or disqualification year-month or the end of data period (May 2020), whichever was earlier.

### Outcome definition

The outcome of the study was the first recorded diagnosis of IHD (including angina pectoris) or stroke event, defined by the presence of diagnosis, hospitalization, and treatment or rehabilitation records. The ICD-10 codes for each outcome are presented in Supplementary Table 1. IHD was considered an event when record of the focal diagnosis (Supplementary Table 1), hospitalization, and treatment occurred in the same month. IHD treatment included percutaneous coronary intervention and coronary artery bypass operations (Supplementary Table 2). Stroke was considered as an event when record of the focal diagnosis (Supplementary Table 1), hospitalization, and treatment occurred in the same month, and any kind of rehabilitation occurred within two months from the diagnosis. Stroke treatment included computed tomography or magnetic resonance imaging followed by medication (antithrombotic agents, ATC code: B01 or edaravone, ATC code: N07X0) or surgical procedures expected to be performed as a part of stroke treatment. In both of the study outcome events, death caused by the focal disease before hospitalization was excluded from the events.

### Predictors

A range of features gathered from annual health check-up records at the index year-month, comprising demographic characteristics, laboratory tests, and questionnaire features were used in the risk prediction model (Supplementary Table 3). Age was available in five-years interval commencing from 30 years, and the median was used as the representative value. For continous features, values beyond the predetermined ranges were considered missing, as they were biologically uncommon and possibly included errors (Supplementary Table 3). Natural log transformation was carried out for all continuous features and dummy encoding for all categorical features. Features with an absolute correlation ≥0.8 were filtered out because including such features often negatively impacts prediction performance. A cut-off value of 0.80 was employed as it is commonly used in regression studies [13].

### Datasets and missing data consideration

To compare prediction models, the study population was divided into the training, validation, and hold-out (testing) sets, as 50%, 25%, and 25%, respectively. For hyperparameter tuning, the training set was used for model development and model performance checked against the validation set. The hold-out set was used for performance checking and model interpretation after model finalization.

Regression models such as Cox and Cox penalized regression using elastic-net (Elnet-Cox) work effectively with complete case records, i.e. without missing values. In a practical use case scenario when performing prediction based on the model, some features may not be available for users. Aiming for such use case scenario, an imputation model was built from the training and validation datasets, and its performance was examined specifically for Cox and Elnet-Cox models. A bagged tree was employed as the imputation model, and a number of trees in the model were explored to obtain reasonable results during the tuning process. The imputation model was built separately for each feature containing missing entries. The bagged tree was used as the imputation model, as it required fewer trees as compared to the random forest method, thereby reducing the computation time [14].

### Prediction models and performance evaluation

Four prediction models were analyzed in the present study: Cox, Elnet-Cox, XGBoost, and Ensemble (average predictions of the Elnet-Cox and XGBoost).

Hyperparameters were tuned through grid search to regulate model complexities. The hyperparameter for Cox and Elnet-Cox was the number of trees in the imputation model. For Elnet-Cox, additional hyperparameters of total amount of regularization (L1 + L2) and proportion of lasso (L1) penalty were considered. Hyperparameters for XGBoost were learning rate, number of trees, maximum depth of the tree, minimum loss reduction to make further partition on a leaf, minimum number of samples in a leaf, fraction of features used in a tree, and fraction of samples used in a tree.

The accuracy of the prediction model was assessed in terms of discrimination performance and calibration. Discrimination was estimated using the Harrell’s C-index [15]. Calibration was visually assessed by calibration line, using the procedure suggested by Crowson et al [16]. Since baseline hazard functions required to draw the calibration lines were not available from the modeling programs (glmnet and XGBoost), they were separately computed using the Breslow baseline hazard estimator [17].

### Model interpretation

Interpretation of the study models was performed through feature importance, accumulated local effects (ALE), [18] and SHapley Additive exPlanations (SHAP) [19]. Of note, 30% of the testing sets were randomly sampled for the model interpretation. Feature importance was obtained by computing the ratio of change in the C-index calculated with permutating each feature. ALE showed how the feature values affect model prediction by computing differences in the predicted values among adjacent observations. SHAP values shows average contribution of features to each instance of prediction, and summing up the SHAP values for all features indicated the contribution to a particular prediction.

### Analyses software

All statistical analyses in the study were performed using R (version 4.0.2) and packages including survival (version 3.2.3) [20], glmnet (version 2.0.18) [21], and XGBoost (version 1.1.1.1) [22].

## Results

### Data extraction

**Fig. 1:**
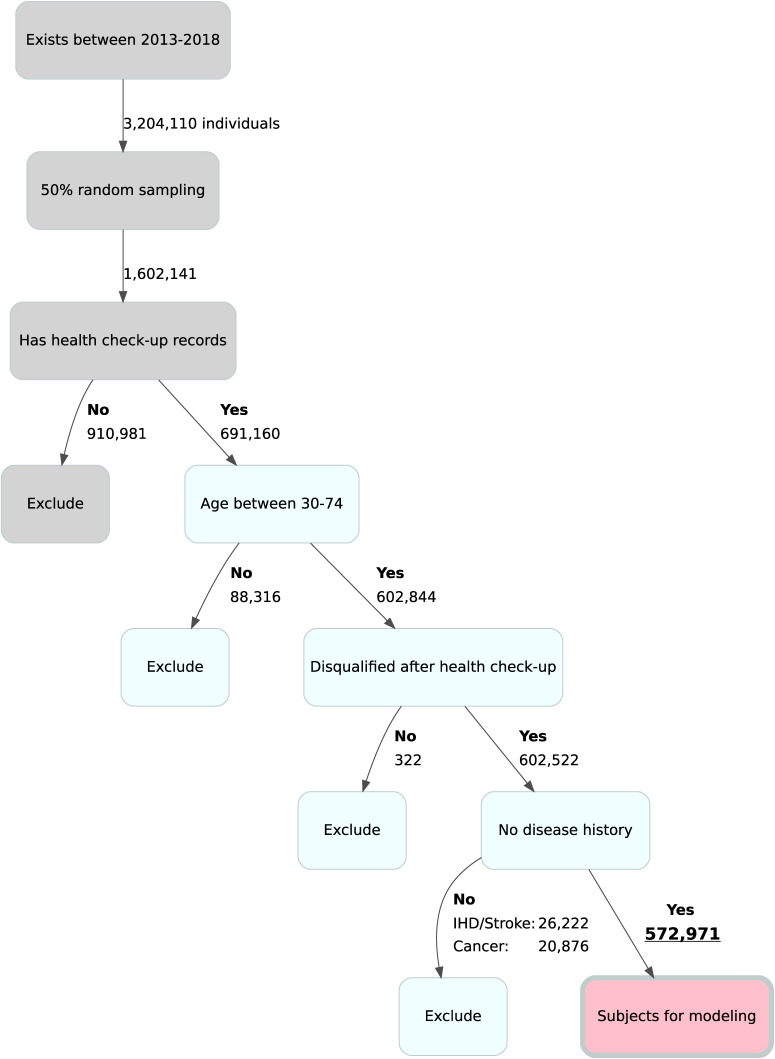
Data extraction IHD: Ischemic heart disease

A total of 1,602,141 individuals were randomly sampled from 3.2 million individuals from the IQVIA Claims Database available during the identification period. After excluding individuals without health check-up records, age <30 or >74 years, disqualified at health check-up, with history of IHD, stroke and/or cancer, the analysis cohort consisted of 572,971 participants (Fig. 1).

### Study population characteristics

**Fig. 2:**
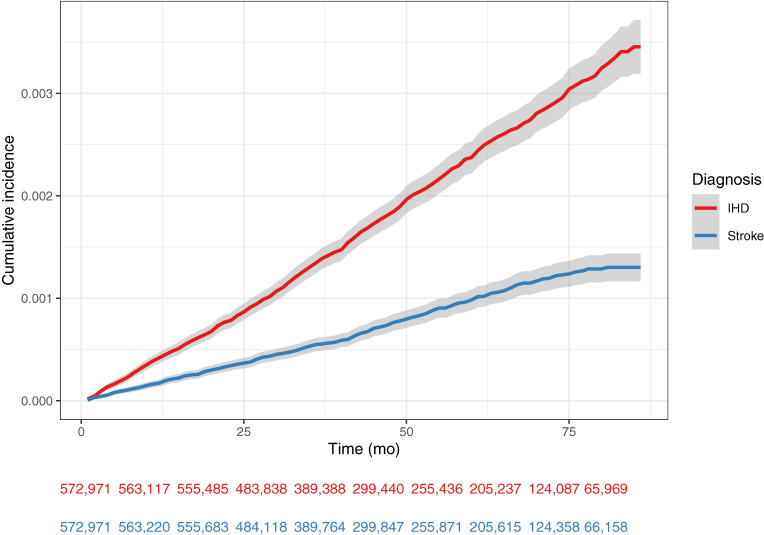
Cumulative incidence of the outcome IHD: Ischemic heart disease; mo: Months Y-axis (Cumulative incidence) equals to 1 − survival probability. Number of individuals at risk for each diagnosis was shown below the figure.

The study population comprised 54% male and 46% female participants. After a median of 48 months follow-up time (for both IHD and stroke), we identified 1,146 IHD and 466 stroke cases. Creatinine and uric acid had missing values in more than half of the records, and were excluded from the features for regression-based models (Cox and Elnet-Cox). Diastolic blood pressure and abdominal circumference were excluded from the features for all models because of their high correlation with other features (systolic blood pressure and body weight; data not shown). Records were removed due to incompleteness from the training set for the Cox (IHD: 16%, stroke: 18%) and Elnet-Cox (69%) models. The study population characteristics are elaborated in Supplementary Table 4. The incidence rates of IHD and stroke were 4.69 and 1.91 per 10,000 person-years, respectively (Fig. 2).

### Model performance

#### Discrimination

Overall, all prediction models showed similarly good performance. The Harrell’s C-index of all IHD models was close to 0.9, which is considered excellent discrimination (0.8–0.9) in binary discrimination problems [23]. For IHD, all prediction models showed similar performance when tested against the imputed (except XGBoost), complete case (except XGBoost), and original (XGBoost only) data. The Harrell’s C-index of stroke models was above 0.7, which is considered an acceptable discrimination (0.7–0.8) [23]. For stroke, all modeling methods showed similar performance but with slight improvement when tested against the imputed data than complete case data in Cox and Elnet-Cox models. The discrimination matrix for IHD and stroke is presented in Table 1.

**Table 1:** Discrimination metrics (Harrell’s C) for IHD and stroke

**Model**	**IHD**		**Stroke**	
	
	**Complete case**	**Imputed (original)**	**Complete case**	**Imputed (original)**
Ensemble	-	0.896	-	0.745
Elnet-Cox	0.913	0.894	0.708	0.734
XGBoost	-	0.893	-	0.737
Cox	0.899	0.894	0.739	0.748

IHD: Ischemic heart disease, XGBoost: Extreme gradient boost

#### Calibration

Among the IHD models, the Cox and the XGBoost was well calibrated when tested against the imputed (or original) data, while Elnet-Cox and Ensemble were off-diagonal for the group with the highest predicted risk when tested against the imputed (or original) data. For complete case data, the Cox and Elnet-Cox models were calibrated, however, with large 95% confidence intervals (CI)—especially among low-risk groups in Elnet-Cox.

**Fig. 3:**
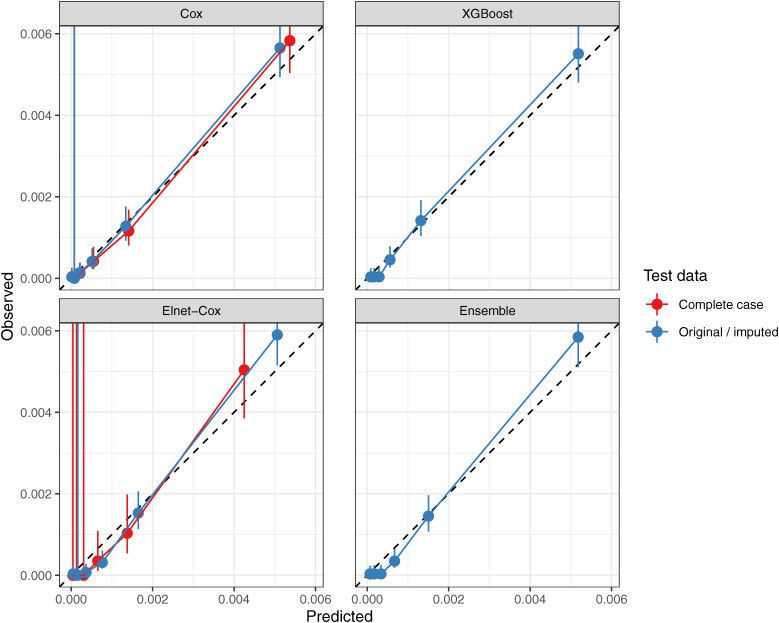
Calibration plot of IHD models Calibration plot showning observed vs. predicted risk of the model. Observations were divided into groups of roughly same size based on the risk score (linear predictor) computed from the model. The observed risk and its 95% confidence intervals were computed from the Poisson GLM.

**Fig. 4:**
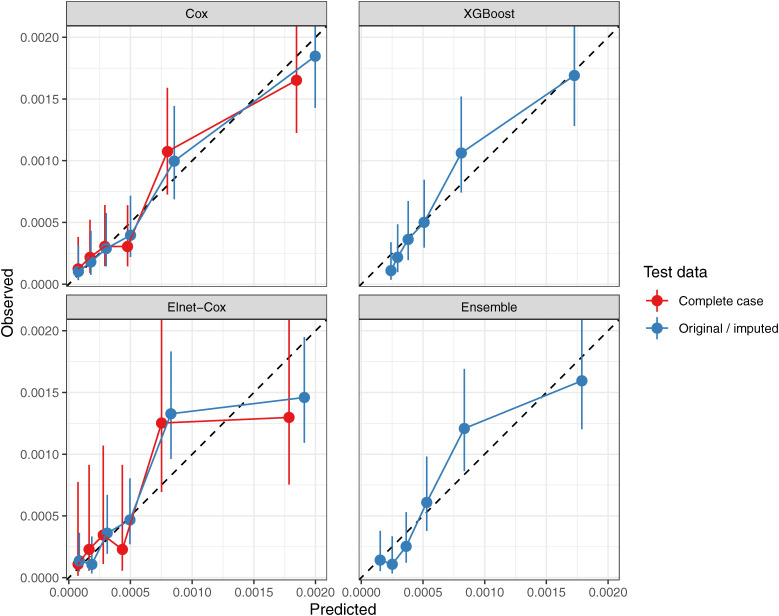
Calibration plot of the stroke models Calibration plot showning observed vs. predicted risk of the model. Observations were divided into groups of roughly same size based on the risk score (linear predictor) computed from the model. The observed risk and its 95% confidence intervals were computed from the Poisson GLM.

Among the stroke models, the Cox and the XGBoost were calibrated when tested against the imputed (or original) data, although with larger CIs than IHD models. When tested against the complete case data, both the Cox and Elnet-Cox models looked calibrated although the CIs were very large. The calibration plots for IHD and stroke models are presented in Figs. 3 and 4.

### Model interpretation

#### Feature importance

**Fig. 5:**
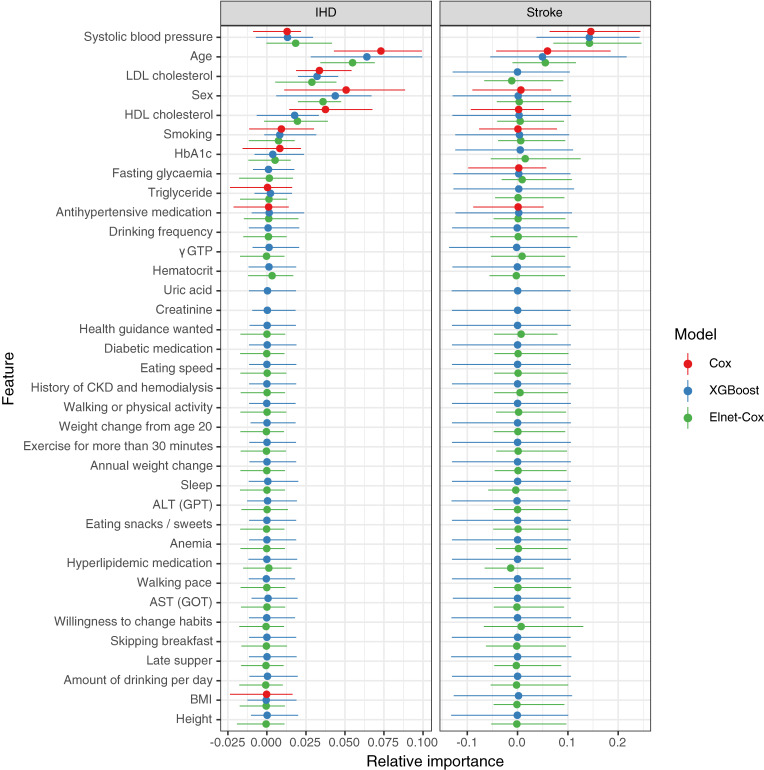
Feature importance for IHD and stroke models AST: Aspartate transaminase; ALT: Alanine transaminase; BMI: Body mass index; CKD: Chronic kidney disease; Cox: Cox proportional-hazards model; HbA_1c_: Glycosalted hemoglobin; GPT: Glutamate pyruvate transaminase; GOT: Glutamate oxalacetate transaminase; HDL: High-density lipoprotein; LDL: Low-density lipoprotein; XGBoost: Extreme gradient boost. Feature was arranged by the mean importance score accorss the models in descending order.

The feature importance for IHD and stroke models, in terms of relative change of the Harrell’s C-index computed from ten permutation samples was shown in Fig. 5. For IHD models, feature importance was generally consistent among the various models but exhibited minor variation in age, sex and high-density-lipoprotein (HDL) cholesterol. Age, sex and HDL cholesterol had higher importance in the Cox model, relative to other models. Body mass index (BMI), antihypertensive medication, and triglyceride, included as features for the Cox model, contributed lesser than the other features.

In contrast, the stroke models relied on fewer features than the IHD models. Systolic blood pressure (SBP) and age were the most important features in stroke models, and their importance was simlar across different stroke models.

#### Accumulated local effects

**Fig. 6:**
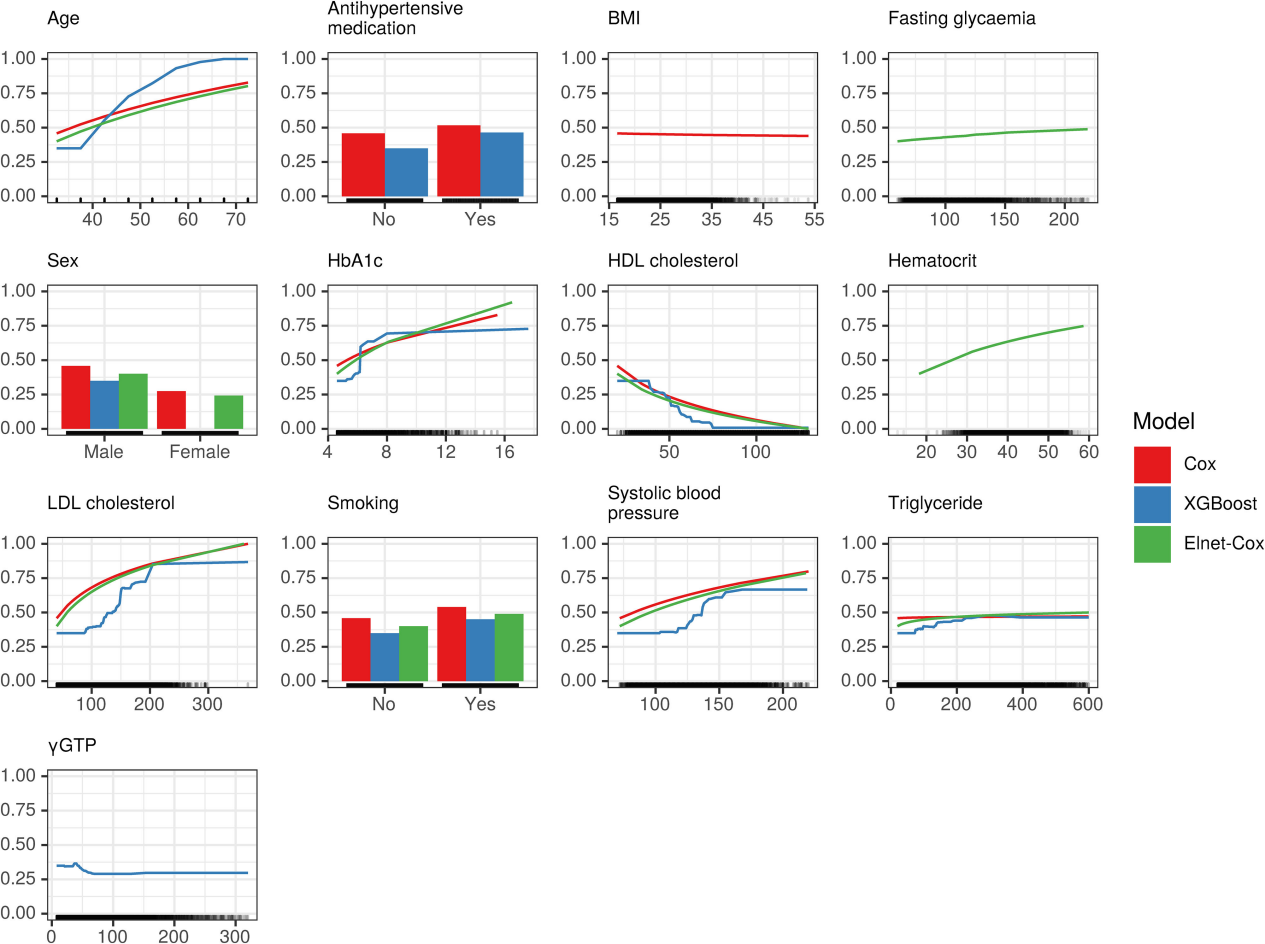
Accumulated local effects plots for IHD model BMI: Body mass index; Cox: Cox proportional-hazards model; HbA_1c_: Glycosalted hemoglobin; γGTP: Gamma-glutamyl transpeptidase; HDL: High-density lipoprotein; LDL: Low-density lipoprotein; XGBoost: Extreme gradient boost. Only top 10 important features were included in the figure.

**Fig. 7:**
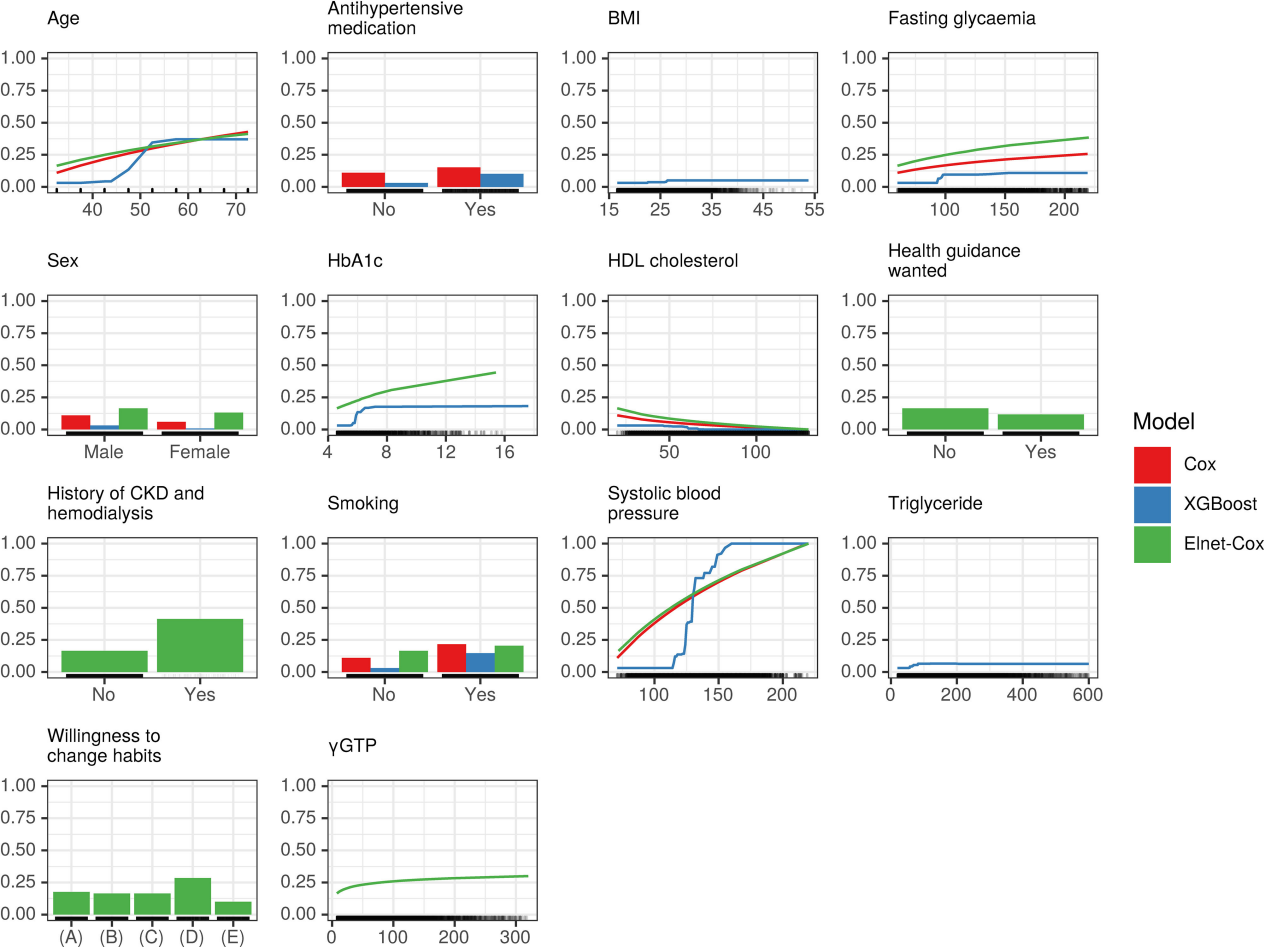
Accumulated local effects plots for stroke model AST: Aspartate transaminase; ALT: Alanine transaminase; BMI: Body mass index; Cox: Cox proportional-hazards model; HbA_1c_: Glycosalted hemoglobin; HDL: High-density lipoprotein; XGBoost: Extreme gradient boost. Only top 10 important features were included in the figure.

The ALE plots for IHD and stroke models are presented in Figs. 6 and 7. For IHD models, the ALE plots for Cox and the Elnet-Cox showed curved lines, as linear regression model inputs include log-transformed features. In contrast, the ALE plots for XGBoost looked nonlinear, as it is a tree-based model. In some features in the XGBoost model, the predicted risk increased or decreased between a limited range and appeared as “S-shape”. For example, the predicted risk increased rapidly between 35 and 60 years of age, while for glycosylated hemoglobin (HbA_1c_), the predicted risk increased between a limited range of around 6%. For SBP, the prediction only increased between 100 and 150 mmHg. Small effect was observed on the prediction at HDL cholesterol levels above 75 mg/dL and at low-density-lipoprotein (LDL) cholesterol levels above 200 mg/dL.

For stroke models, the ALE plots varied, but there were similarities between the Cox and Elnet-Cox models for SBP. The ALE plots for XGBoost were nonlinear, and appeared distinct from other models. As per the ALE plot for XGBoost, the predicted risk increased rapidly between 35 and 55 years of age and between an SBP of 120 and 160 mmHg.

#### SHAP

The SHAP plots suggest that the nonlinear effects were not a result of lack of observation. The SHAP dependence plots for the IHD and stroke models showed similar tendency with the ALE plots (Supplementary Figures 1 and 2).

## Discussion

The present study implemented statistical regression and ML methods to develop a prognostic model for IHD and stroke, based on large-scale real-world database of general population. In consistency with previous traditional cohort studies [8, 24–26], SBP and age were identified as the most crucial features in all stroke models. For IHD models, LDL, HDL, gender, HbA_1c_, and smoking history also contributed significantly to risk prediction besides SBP and age—similar to previous studies in Japan [5, 8, 24].

Compared to the ML model, the Cox model showed similar discrimination accuracy despite having a smaller training dataset. The authors acknowledge that a direct comparison of the discrimination metric with previous studies in Japanese population is not feasible, owing to differences in study follow-up periods, nature of the data, and outcomes definitions. Nevertheless, the discrimination scores of this study were analogous to previous prognostic models for Japanese populations [5, 6, 8]. For calibration accuracy, 95% CI of the complete case data in the Elnet-Cox model was large, particularly in low-risk groups, because of a smaller sample size than Cox model, owing to the larger number of features and fewer complete case records. The calibration plots for IHD models (Fig. 3) indicate that the models tend to over-estimate in the highest risk group and therefore must be carefully interpreted. The imputation model did not worsen the discrimination accuracy and the calibraion.

From a clinical perspective, investigating a nonlinear relationship found in the ALE and the SHAP plots between features and outcomes might be of interest: the risk of IHD events decreased linearly with HDL below 75 mg/dL, but was static over 75 mg/dL (Fig. 6 and Supplementary Figure 1). There are controversial evidence regarding the association between cardiovascular disease and high HDL. Interestingly, our result was not consistent with a pooled analysis of Japanese cohort studies [25], which showed higher cardiovascular disease risk in individuals with very high HDL (≥90 mg/dL). The S-shape curve of SBP observed in both models can be attributed to similar predicted risks for subjects with SBP 150–160 mmHg and higher. Enrollees with abnormally high blood pressure might be censored from the dataset due to various reasons—including death. Further research is imperative to assess whether the “S-shape” in the XGBoost model found in present study remains consistent after controlling covariates.

The features in the Cox model were risk factors reported in previous studies [5, 6, 8], and the majority were mandatory tests for the annual health check-up. Therefore, the features in the Cox model can be imposed to widespread use. The Cox model can be conveniently adapted for system development, as the event risk can be computed by simple arithmetic calculation. However, in practice, the imputation process is crucial for Cox models, as it promotes accessibility to missing records, thus enabling broader implementation. For example, fasting glycemia may be entered as a missing value if individuals may have eaten a meal before the exam. Therefore, system development and management for Cox models require both prediction and imputation models. In contrast, the XGBoost model is advantageous over Cox model, as it accepts missing values and does not require the imputation process.

In many cases, the health check-up results are generated as printed hard copies for individuals, and electronic records are not utilized. Use of electronic records, which typically include additional features, will be preferable for the ML-based risk prediction models. The best scenario to implement the ML-based prediction model will be building this as a part of personal health record management system, which includes health check-up records and other individuals backgrounds.

The output from the prediction model should be interpretated as a relative risk rather than an absolute event probability, because an association between cardiovascular events and surrounding environment has been reported. The cardiovascular events were linked with seasonal and regional variations in temperature [27]. An association between room temperature and biomarkers for the cardiovascular events including high blood pressure and electrocardiogram abnormality were also reported [28]. In practical use, predicted relative risk and risk factors based on the model may be reported back to users, which may include individuals who took a medical check-up, medical facilities that provide medical check-ups, public health nurses, and health insurance providers. The predicted risk and risk factors may be used for consultation and guidance to lead a healthier lifestyle, including an advice on appropriate room temperature.

In the present study, the incidence rates of IHD and stroke events were lower than those reported in previous studies in Japan [24]. In addition, contrary to previous reports in Japan, the incidence rate of IHD was higher than that of stroke. Kitamura *et al.* [24], estimated age-adjusted IHD incidence rates (per 10,000 person-years) for the Japanese population aged 40–69 years to be around 7–13 in males and two in females in the last two decades. Previous studies have estimated age-adjusted stroke incidence rates of around 30–40 per 10,000 person-years in the last few decades [2, 29, 30], which is about fifteen times higher than those reported in the present study. Considerably older population of previous studies than this study can be associated to lower incidence rates of IHD and stroke with present study. Furthermore, the outcome definition of this study excludes severe and fatal cases. The reversal of incidence rates in the present study may be attributed to the difference in the susceptible age between the diseases. The susceptible age of stroke was higher than that of IHD, and incidence rates of the two diseases were much more similar in ages <60 than >70 [26]. Therefore, it is possible that the incidence rates remain the same or reverse as the present study, in the younger population.

Despite the predictive accuracy of the prognostic models in the present study, the model should be interpreted in the context of its limitations. In contrast to previous studies [5–8], which included individuals residing in certain regions, majority of individuals in the IQVIA claims data were workers in the secondary and tertiary industries across Japan. The database lacks records for the elderly population (>75 years of age). Therefore, prediction models obtained from current and previous studies should be interpreted with caution. The model can be utilized to formulate preventive measures for the younger population, particularly the working generation. Additional validation using external data from different health insurance providers which may include individuals with different backgrounds will give more credibility to the model prediction. Further research is required to develop prognostic models for other population groups such as the elderly. The study cohort was derived from an administrative claims database, which incorporates unvalidated outcomes. The outcomes definition in the study was prudently selected through sensitivity analyses, considering the characteristic of the data source. However, the connection with actual events was unknown. As written in the outcome definition section, a death caused by the focal disease before hospitalization was excluded from the events. Death is considered a competing event, but it was not explicitly captured in the database.

## Conclusion

The current study explored an established regression model, as well as ML algorithms approach for risk prediction of IHD and stroke, based on a large-scale real-world database from Japan. Considering that each model differs in its characteristics, it is prudent to assess various approaches. For IHD models, the ALE plots for Cox and the Elnet-Cox showed curved lines, whereas it appeared as “S-shape” for XGBoost model. For stroke models, the ALE plots for XGBoost were nonlinear and distinct from other models. The study identified SBP and age as primary risk predictors for all stroke models. Of note, LDL, HDL, gender, HbA_1c_, and smoking history also contributed significantly to risk prediction for IHD besides SBP and age. The study provides a robust framework for large database applications and obtain social implementation of risk prediction models in Japan. Further utilizing the framework, it is able to iteratively refine the developed risk prediction modes, and construct risk prediction models targeting other disease and clinical outcomes.

## Abbreviations

ALE: Accumulated local effects
ALT: Alanine transaminase
BMI: Body mass index
CI: Confidence intervals
CKD: Chronic kidney disease
GOT: Glutamate oxalacetate transaminase
GPT: Glutamate pyruvate transaminase
HDL: High-density-lipoprotein
LDL: Low-density-lipoprotein
ML: Machine learning
RWD: Real-world data
SBP: Systolic blood pressure
SHAP: SHapley Additive exPlanations

## Supplementary information

**Additional file 1:** **Figure 1:** SHAP dependance plots for IHD Models. **Figure 2:** SHAP dependance plots for Stroke models. **Supplementary Table 1:** List of ICD-10 codes. **Supplementary Table 2:** Treatment code for the outcome definition. **Supplementary Table 3:** Risk Variables. **Supplementary Table 4:** Study population characteristics.

## Declarations

### Availability of data and material

The data used to develop and validate the predictive model are commercially available. The developed model is not publicly available.
