# Supplementary material for: Development and validation of ischemic heart disease and stroke prognostic models using large-scale real-world data from Japan
Source: Environ Health Prev Med. 2023 Feb 15;28:16. doi: 10.1265/ehpm.22-00106 (PMC9989775; doi:10.1265/ehpm.22-00106)
Supplement: Supplementary file 1 — Additional file 1: Figure 1: SHAP dependance plots for IHD Models. Figure 2: SHAP dependance plots for Stroke models. Supplementary Table 1: List of ICD-10 codes. Supplementary Table 2: Treatment code for the outcome definition. Supplementary Table 3: Risk Variables. Supplementary Table 4: Study population characteristics. [file ehpm-28-016-s001.docx]

Supplementary Figure

Figure 1: SHAP dependance plots for IHD Models


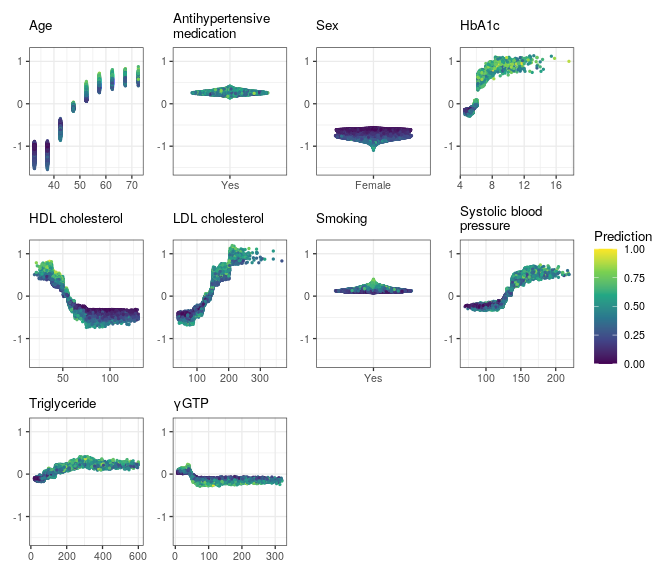


HbA_1c_: Glycosalted hemoglobin; γGTP: Gamma-glutamyl transpeptidase; HDL: High-density lipoprotein; IHD: Ischemic heart disease; LDL: Low-density lipoprotein.

Only top 10 important features were included in the figure

FIGURE 2: SHAP dependance plots for Stroke models


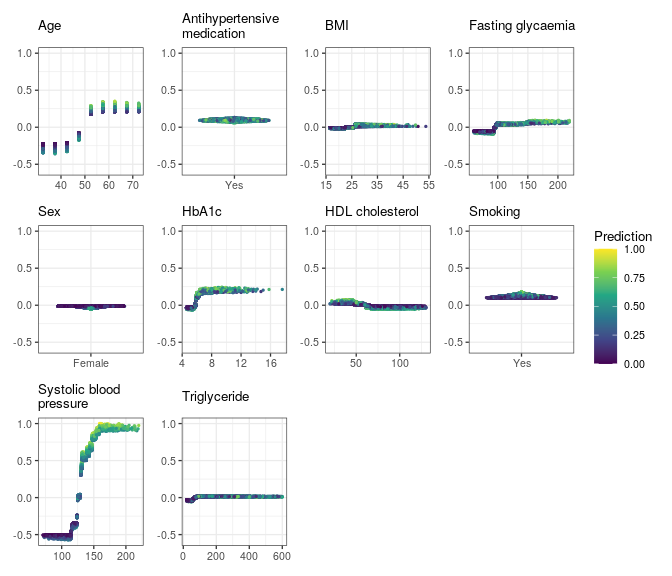


BMI: Body mass index; HbA_1c_: Glycosalted hemoglobin.

Only top 10 important features were included in the figure

**SUPPLEMENTARY TABLES**

Supplementary Table 1: List of ICD-10 codes

| Diagnosis | ICD10 | Description | Exclude |
| --- | --- | --- | --- |
| IHD | I200 | Unstable angina |  |
| IHD | I201 | Angina pectoris with documented spasm | Variant angina, vasospastic angina |
| IHD | I208 | Other forms of angina pectoris |  |
| IHD | I209 | Angina pectoris, unspecified |  |
| IHD | I210 | Acute transmural myocardial infarction of anterior wall |  |
| IHD | I211 | Acute transmural myocardial infarction of inferior wall |  |
| IHD | I212 | Acute transmural myocardial infarction of other sites |  |
| IHD | I213 | Acute transmural myocardial infarction of unspecified site |  |
| IHD | I214 | Acute subendocardial myocardial infarction |  |
| IHD | I219 | Acute myocardial infarction, unspecified |  |
| IHD | I240 | Coronary thrombosis not resulting in myocardial infarction |  |
| IHD | I241 | Dressler syndrome |  |
| IHD | I248 | Other forms of acute ischemic heart disease | Coronary artery dissection, coronary insufficiency |
| IHD | I249 | Acute ischemic heart disease, unspecified |  |
| Stroke | I600 | Subarachnoid hemorrhage from carotid siphon and bifurcation |  |
| Stroke | I601 | Subarachnoid hemorrhage from middle cerebral artery |  |
| Stroke | I602 | Subarachnoid hemorrhage from anterior communicating artery |  |
| Stroke | I603 | Subarachnoid hemorrhage from posterior communicating artery |  |
| Stroke | I604 | Subarachnoid hemorrhage from basilar artery |  |
| Stroke | I605 | Subarachnoid hemorrhage from vertebral artery |  |
| Stroke | I606 | Subarachnoid hemorrhage from other intracranial arteries |  |
| Stroke | I607 | Subarachnoid hemorrhage from intracranial artery, unspecified |  |
| Stroke | I608 | Other subarachnoid hemorrhage |  |
| Stroke | I609 | Subarachnoid hemorrhage, unspecified |  |
| Stroke | I610 | Intracerebral hemorrhage in hemisphere, subcortical |  |
| Stroke | I611 | Intracerebral hemorrhage in hemisphere, cortical |  |
| Stroke | I613 | Intracerebral hemorrhage in brain stem |  |
| Stroke | I614 | Intracerebral hemorrhage in cerebellum |  |
| Stroke | I615 | Intracerebral hemorrhage, intraventricular |  |
| Stroke | I616 | Intracerebral hemorrhage, multiple localized |  |
| Stroke | I618 | Other intracerebral hemorrhage |  |
| Stroke | I619 | Intracerebral hemorrhage, unspecified |  |
| Stroke | I630 | Cerebral infarction due to thrombosis of precerebral arteries |  |
| Stroke | I631 | Cerebral infarction due to embolism of precerebral arteries |  |
| Stroke | I632 | Cerebral infarction due to unspecified occlusion or stenosis of precerebral arteries |  |
| Stroke | I633 | Cerebral infarction due to thrombosis of cerebral arteries |  |
| Stroke | I634 | Cerebral infarction due to embolism of cerebral arteries |  |
| Stroke | I635 | Cerebral infarction due to unspecified occlusion or stenosis of cerebral arteries |  |
| Stroke | I636 | Cerebral infarction due to cerebral venous thrombosis, nonpyrogenic |  |
| Stroke | I638 | Other cerebral infarction |  |
| Stroke | I639 | Cerebral infarction, unspecified |  |
| Stroke | I64 | Stroke, not specified as hemorrhage or infarction |  |

IHD: Ischemic heart disease

Supplementary Table 2: Treatment code for the outcome definition

| Diagnosis | Code | Description |
| --- | --- | --- |
| IHD | K546 | Percutaneous transluminal coronary angioplasty |
| IHD | K547 | Percutaneous coronary plaque removal |
| IHD | K548 | Percutaneous transluminal coronary angioplasty with sppecial catheter |
| IHD | K549 | Percutaneous transluminal coronary angioplasty with stent insertion |
| IHD | K550 | Coronary thrombolytic therapy |
| IHD | K551 | Coronary angioplasty:thromboendarterectomy |
| IHD | K552 | Coronary artery bypass grafting |
| IHD | K553 | Surgical resection of ventricular aneurysm |
| Stroke | E200 | Computed tomography: procedure |
| Stroke | E201 | Nonradioactive xenon inspection of cerebral blood flow state |
| Stroke | E202 | Magnetic resonance imaging |
| Stroke | E203 | Computed image diagnosis |
| Stroke | K145 | External ventricular drain |
| Stroke | K146 | Strip craniectomy |
| Stroke | K147 | Trepanation |
| Stroke | K148 | Testing craniectomy |
| Stroke | K149 | Decompressive craniectomy |
| Stroke | K160 | Brain surgery |
| Stroke | K163 | Subdural hematoma removal |
| Stroke | K164 | Intracranial hematoma removal |
| Stroke | K167 | Intracranial mass removal |
| Stroke | K168 | Brain resection |
| Stroke | K174 | Surgery for Hydrocephalus |
| Stroke | K175 | Cerebral aneurysm wrapping |
| Stroke | K176 | Cerebral aneurysm clipping: inflow vessel |
| Stroke | K177 | Cerebral aneurysm clipping: neck |
| Stroke | K178 | Endovascular brain surgery |
| Stroke | K179 | Surgery for cerebrospinal fluid leakage |
| Stroke | K610 | Angioplasty, anastomosis |
| Stroke | K615 | Vessel embolization |
| Stroke | H000-H008 | Rehabilitation |

Supplementary Table 3: Risk Variables

| Continuos risk variables | | |
| --- | --- | --- |
| **Feature** | **Lower limit** | **Upper limit** |
| Height (m) | 1 | 2 |
| Weight (kg) | 39 | 150 |
| BMI | 16.6 | 60 |
| Abdominal circumference (cm) | 50 | 170 |
| HbA_1c_ (%) | 4.6 | 23 |
| Fasting glycemia (mg/dL) | 60 | 220 |
| Hematocrit (%) | 0 | 90 |
| HDL cholesterol (mg/dL) | 20 | 130 |
| LDL cholesterol (mg/dL) | 40 | 400 |
| Triglycerides (mg/dL) | 20 | 600 |
| Systolic blood pressure (mmHg) | 70 | 220 |
| Diastolic blood pressure (mmHg) | 45 | 180 |
| GOT (AST) (U/L) | 10 | 1000 |
| GPT (ALT) (U/L) | 6 | 1000 |
| Gamma GTP(U/L) | 8 | 320 |
| Uric acid (mg/dL) | 2 | 12 |
| Creatinine (mg/dL) | 0 | 25 |
| **Questionnaire** | | |
| **Feature** | **Typical question** | |
| Smoking history | Do/Did you smoke regularly? | |
| Eating speed | Do you eat faster than others? | |
| Late supper | Do you often eat within 2 hours before going to bed? | |
| Eating snacks / sweets | How often you eat snacks / sweets? | |
| Skipping breakfast | Do you skip breakfast more than three times a week? | |
| Drinking habit | How often you drink alcohol? | |
| Drinking amount | How much you drink per day? | |
| Sleep | Do you sleep well? | |
| Walking | Do you walk or do other activities at least 1 hour every day? | |
| Waking pace | Do you walk faster than others in your age? | |
| Exercise | Do you regularly exercise at least twice a week this year? | |
| Weight change from last year | Did your weight change since last year? | |
| Weight change from age 20 | Are you heavier than when you were 20 years old? | |
| History of CKD and hemodialysis | Have you ever diagnosed chronic kidney diseases, or took hemodialysis treatment? | |
| Antihypertensive medication | Are you taking antihypertensive medication? | |
| Diabetic medication | Are you taking diabetic medication? | |
| Hyperlipidemic medication | Are you taking hyperlipidemic medication? | |
| Willingness to change habits | Are you willing to improve your eating / exercise habits? | |

AST: Aspartate transaminase; ALT: Alanine transaminase; BMI: Body mass index; CKD: Chronic kidney disease; HbA_1c_: Glycosalted hemoglobin; GPT: Glutamate pyruvate transaminase; GOT: Glutamate oxalacetate transaminase; HDL: High-density lipoprotein; LDL: Low-density lipoprotein

Supplementary Table 4: Study population characteristics

| **Variables** | **Parameters** | **Overall** | **IHD** | **Stroke** |
| --- | --- | --- | --- | --- |
| Sex | N | 572,971 | 1,146 | 466 |
|  | Complete rate | 1.00 | 1.00 | 1.00 |
|  | Male, n (%) | 307,500 (54) | 1,056 (92) | 344 (75) |
|  | Female, n (%) | 265,471 (46) | 90 (8) | 122 (25) |
| Age | N | 572,971 | 1,146 | 466 |
|  | Complete rate | 1.00 | 1.00 | 1.00 |
|  | Mean (SD) | 46.46 (9.45) | 54.28 (7.58) | 52.66 (8.41) |
|  | Median (Min, Max) | 47.50 (32.50,72.50) | 52.50 (32.50,72.50) | 52.50 (32.50,72.50) |
| Height | N | 567,566 | 1,141 | 463 |
|  | Complete rate | 0.99 | 1.00 | 0.99 |
|  | Mean (SD) | 1.65 (0.09) | 1.68 (0.07) | 1.66 (0.08) |
|  | Median (Min, Max) | 1.65 (1.05, 2.00) | 1.69 (1.42, 1.89) | 1.67 (1.40, 1.87) |
| Weight | N | 567,879 | 1,141 | 463 |
|  | Complete rate | 0.99 | 1.00 | 0.99 |
|  | Mean (SD) | 62.85 (13.03) | 71.78 (12.32) | 67.99 (13.22) |
|  | Median (Min, Max) | 61.50 (39.00, 150.00) | 70.80 (39.50, 130.30) | 66.95 (42.00, 139.90) |
| BMI | N | 562,493 | 1,138 | 457 |
|  | Complete rate | 0.98 | 0.99 | 0.98 |
|  | Mean (SD) | 22.96 (3.75) | 25.20 (3.53) | 24.48 (3.83) |
|  | Median (Min, Max) | 22.40 (16.60, 59.80) | 24.80 (16.80, 44.00) | 23.90 (17.20, 46.10) |
| Abdominal circumference | N | 537,698 | 1,138 | 459 |
|  | Complete rate | 0.94 | 0.99 | 0.98 |
|  | Mean (SD) | 81.32 (10.18) | 88.00 (9.33) | 85.61 (10.17) |
|  | Median (Min, Max) | 80.50 (50.00, 168.00) | 87.00 (60.20, 134.30) | 85.00 (52.80, 137.50) |
| HbA_1c_ | N | 500,226 | 1,008 | 411 |
|  | Complete rate | 0.87 | 0.88 | 0.88 |
|  | Mean (SD) | 5.51 (0.61) | 618 (1.28) | 5.89 (1.18) |
|  | Median (Min, Max) | 5.40 (4.60, 17.60) | 5.70 (4.60, 14.20) | 5.60 (4.60, 12.40) |
| Fasting glycemia | N | 476,641 | 880 | 361 |
|  | Complete rate | 0.83 | 0.77 | 0.77 |
|  | Mean (SD) | 93.96 (14.41) | 109.14 (25.72) | 101.29 (19.42) |
|  | Median (Min, Max) | 92.00 (60.00, 220.00) | 102.00 (73.00, 220.00) | 98.00 (69.00, 219.00) |
| Hematocrit | N | 394,134 | 706 | 301 |
|  | Complete rate | 0.69 | 0.62 | 0.65 |
|  | Mean (SD) | 42.80 (4.31) | 45.68 (3.68) | 43.93 (4.21) |
|  | Median (Min, Max) | 43.00 (1.00, 84.00) | 45.90 (30.10, 59.40) | 44.00 (29.30, 56.40) |
| HDL | N | 552,858 | 1,139 | 459 |
|  | Complete rate | 0.96 | 0.99 | 0.98 |
|  | Mean (SD) | 64.23 (16.62) | 51.10 (13.00) | 59.35 (15.60) |
|  | Median (Min, Max) | 62.00 (20.00, 130.00) | 49.00 (23.00, 121.00) | 57.00 (29.00, 122.00) |
| LDL | N | 553,096 | 1,137 | 458 |
|  | Complete rate | 0.97 | 0.99 | 0.98 |
|  | Mean (SD) | 120.69 (31.42) | 141.92 (34.11) | 128.74 (33.09) |
|  | Median (Min, Max) | 118.00 (40.00, 396.00) | 141.00 (51.00, 275.00) | 127.50 (42.00, 233.00) |
| Triglyceride | N | 551,182 | 1,126 | 455 |
|  | Complete rate | 0.96 | 0.98 | 0.98 |
|  | Mean (SD) | 104.28 (71.33) | 162.23 (91.91) | 129.20 (76.07) |
|  | Median (Min, Max) | 83.00 (20.00, 600.00) | 141.00 (29.00, 586.00) | 108.00 (26.00, 444.00) |
| SBP | N | 570,716 | 1,145 | 466 |
|  | Complete rate | 1.00 | 1.00 | 1.00 |
|  | Mean (SD) | 119.03 (16.97) | 131.99 (18.47) | 134.45 (20.08) |
|  | Median (Min, Max) | 118.00 (70.00, 220.00) | 130.00 (83.00, 220.00) | 132.00 (82.00, 220.00) |
| DBP | N | 569,718 | 1,145 | 466 |
|  | Complete rate | 1.00 | 1.00 | 1.00 |
|  | Mean (SD) | 73.84 (12.09) | 82.84 (12.46) | 84.81 (14.20) |
|  | Median (Min, Max) | 73.00 (45.00, 180.00) | 82.00 (46.00, 145.00) | 84.00 (51.00, 160.00) |
| AST (GOT) | N | 554,390 | 1,134 | 461 |
|  | Complete rate | 0.97 | 0.99 | 0.99 |
|  | Mean (SD) | 22.28 (11.08) | 24.54 (12.04) | 25.21 (14.62) |
|  | Median (Min, Max) | 20.00 (10.00, 988.00) | 22.00 (11.00, 148.00) | 22.00 (10.00, 174.00) |
| ALT (GPT) | N | 492,197 | 912 | 373 |
|  | Complete rate | 0.86 | 0.80 | 0.80 |
|  | Mean (SD) | 22.85 (18.13) | 30.71 (21.72) | 27.78 (20.36) |
|  | Median (Min, Max) | 18.00 (6.00, 914.00) | 25.00 (6.00, 220.00) | 21.00 (7.00, 179.00) |
| γGTP | N | 550,567 | 1,132 | 458 |
|  | Complete rate | 0.96 | 0.99 | 0.98 |
|  | Mean (SD) | 35.19 (34.93) | 47.42 (39.10) | 50.70 (50.78) |
|  | Median (Min, Max) | 23.00 (8.00, 320.00) | 36.00 (8.00, 316.00) | 35.00 (10.00, 318.00) |
| Uric acid | N | 169,269 | 322 | 149 |
|  | Complete rate | 0.30 | 0.28 | 0.32 |
|  | Mean (SD) | 5.33 (1.41) | 6.15 (1.29) | 5.82 (1.32) |
|  | Median (Min, Max) | 5.20 (2.00, 11.90) | 6.15 (2.60, 10.40) | 5.80 (2.50, 9.20) |
| Creatinine | N | 185,389 | 339 | 157 |
|  | Complete rate | 0.32 | 0.30 | 0.34 |
|  | Mean (SD) | 0.75 (0.24) | 0.90 (0.64) | 0.78 (0.18) |
|  | Median (Min, Max) | 0.73 (0.05, 15.58) | 0.84 (0.41, 11.72) | 0.79 (0.40, 1.44) |
| Smoking | N | 564,108 | 1,142 | 459 |
|  | Complete rate | 0.98 | 1.00 | 0.98 |
|  | No, n (%) | 408,833 (72) | 553 (48) | 267 (58) |
|  | Yes, n (%) | 155,275 (28) | 589 (52) | 192 (42) |
| Eating speed | N | 509,058 | 970 | 403 |
|  | Complete rate | 0.89 | 0.85 | 0.86 |
|  | Slower than others, n (%) | 45,483 (9) | 61 (6) | 34 (8) |
|  | On average | 295,217 (58) | 551 (57) | 225 (56) |
|  | Faster than others, n (%) | 168,358 (33) | 358 (37) | 144 (36) |
| Late supper | N | 509,929 | 966 | 403 |
|  | Complete rate | 0.89 | 0.84 | 0.86 |
|  | No, n (%) | 317,719 (62) | 586 (61) | 230 (57) |
|  | Yes, n (%) | 192,210 (38) | 380 (39) | 173 (43) |
| Eating snacks / sweets | N | 506,981 | 966 | 402 |
|  | Complete rate | 0.89 | 0.84 | 0.86 |
|  | No, n (%) | 367,984 (73) | 801 (83) | 313 (78) |
|  | Yes, n (%) | 138,997 (27) | 165 (17) | 89 (22) |
| Skipping breakfast | N | 508,153 | 962 | 402 |
|  | Complete rate | 0.89 | 0.84 | 0.86 |
|  | No, n (%) | 380,308 (75) | 743 (77) | 285 (71) |
|  | Yes, n (%) | 127,845 (25) | 219 (23) | 117 (29) |
| Drinking frequency | N | 516,729 | 976 | 410 |
|  | Complete rate | 0.90 | 0.85 | 0.88 |
|  | Do not drink, n (%) | 203,259 (39) | 427 (44) | 126 (31) |
|  | Few times in a week, n (%) | 189,288 (37) | 321 (33) | 131 (32) |
|  | Everyday, n (%) | 124,182 (24) | 228 (23) | 153 (37) |
| Amount of drinking per day | N | 422,846 | 763 | 334 |
|  | Complete rate | 0.74 | 0.67 | 0.72 |
|  | <180ml / day, n (%) | 217,984 (52) | 353 (46) | 136 (41) |
|  | 180 - 359ml / day, n (%) | 121,954 (29) | 224 (29) | 109 (33) |
|  | 360 - 539ml / day, n (%) | 58,971 (14) | 130 (17) | 69 (21) |
|  | 540+ml / day, n (%) | 23,937 (6) | 56 (7) | 20 (6) |
| Sleep | N | 508,542 | 964 | 401 |
|  | Complete rate | 0.89 | 0.84 | 0.86 |
|  | No, n (%) | 204,941 (40) | 401 (42) | 165 (41) |
|  | Yes, n (%) | 303,601 (60) | 563 (58) | 236 (59) |
| Walking or physical activity | N | 508,960 | 967 | 401 |
|  | Complete rate | 0.89 | 0.84 | 0.86 |
|  | No, n (%) | 297,146 (58) | 611 (63) | 269 (67) |
|  | Yes, n (%) | 211,814 (42) | 356 (37) | 132 (33) |
| Walking pace | N | 507,794 | 961 | 401 |
|  | Complete rate | 0.89 | 0.84 | 0.86 |
|  | No, n (%) | 262,365 (52) | 522 (54) | 215 (54) |
|  | Yes, n (%) | 245,429 (48) | 439 (46) | 186 (46) |
| Exercise for more than 30 minutes | N | 508,457 | 963 | 401 |
|  | Complete rate | 0.89 | 0.84 | 0.86 |
|  | No, n (%) | 413,347 (81) | 775 (80) | 323 (81) |
|  | Yes, n (%) | 95,110 (19) | 188 (20) | 78 (19) |
| Annual weight change | N | 418,775 | 852 | 351 |
|  | Complete rate | 0.73 | 0.74 | 0.75 |
|  | No, n (%) | 292,262 (70) | 604 (71) | 241 (69) |
|  | Yes, n (%) | 126,513 (30) | 248 (29) | 110 (31) |
| Weight change from age 20 | N | 508,115 | 964 | 399 |
|  | Complete rate | 0.89 | 0.84 | 0.86 |
|  | No, n (%) | 338,996 (67) | 452 (47) | 219 (55) |
|  | Yes, n (%) | 169,159 (33) | 512 (53) | 180 (45) |
| History of CKD and hemodialysis | N | 509,247 | 976 | 407 |
|  | Complete rate | 0.89 | 0.85 | 0.87 |
|  | No, n (%) | 507,872 (100) | 971 (99) | 402 (99) |
|  | Yes, n (%) | 1,375 (0) | 5 (1) | 5 (1) |
| Antihypertensive medication | N | 561,770 | 1,141 | 461 |
|  | Complete rate | 0.98 | 1.00 | 0.99 |
|  | No, n (%) | 513,516 (91) | 820 (72) | 352 (76) |
|  | Yes, n (%) | 48,254 (9) | 321 (28) | 109 (24) |
| Diabetic medication | N | 561,722 | 1,142 | 461 |
|  | Complete rate | 0.98 | 1.00 | 0.99 |
|  | No, n (%) | 545,127 (97) | 978 (86) | 419 (91) |
|  | Yes, n (%) | 16,595 (3) | 164 (14) | 42 (9) |
| Hyperlipidemic medication | N | 561,714 | 1,142 | 461 |
|  | Complete rate | 0.98 | 1.00 | 0.99 |
|  | No, n (%) | 535,570 (95) | 971 (85) | 423 (92) |
|  | Yes, n (%) | 26,144 (5) | 171 (15) | 38 (8) |
| Willingness to change habits | N | 506,923 | 964 | 398 |
|  | Complete rate | 0.88 | 0.84 | 0.85 |
|  | Not willing to improve, n (%) | 126,275 (25) | 210 (22) | 88 (22) |
|  | Willing to improve in 6 months, n (%) | 196,045 (39) | 363 (38) | 155 (39) |
|  | Just started improvement, n (%) | 82,888 (16) | 156(16) | 67 (17) |
|  | Improved less than 6 months ago, n (%) | 47,506 (9) | 93 (10) | 46 (12) |
|  | Improved before 6 months ago, n (%) | 54,209 (11) | 142 (15) | 42 (11) |
| Anemia | N | 504,927 | 965 | 402 |
|  | Complete rate | 0.88 | 0.84 | 0.86 |
|  | No, n (%) | 442,323 (88) | 934 (97) | 368 (92) |
|  | Yes, n (%) | 62,604 (12) | 31 (3) | 34 (8) |
| Health guidance wanted | N | 501,535 | 966 | 396 |
|  | Complete rate | 0.88 | 0.84 | 0.85 |
|  | No, n (%) | 314,057 (63) | 608 (63) | 266 (67) |
|  | Yes, n (%) | 187,478 (37) | 358 (37) | 130 (33) |

AST: Aspartate transaminase; ALT: Alanine transaminase; BMI: Body mass index; CKD: Chronic kidney disease; DBP: Diastolic blood pressure; HbA_1c_: Glycosalted hemoglobin; GPT: Glutamate pyruvate transaminase; GOT: Glutamate oxalacetate transaminase; HDL: High-density lipoprotein; LDL: Low-density lipoprotein; SBP: Systolic blood pressure
